# Probabilistic Models to Describe the Dynamics of Migrating Microbial Communities

**DOI:** 10.1371/journal.pone.0117221

**Published:** 2015-03-24

**Authors:** Joanna L Schroeder, Mary Lunn, Ameet J Pinto, Lutgarde Raskin, William T Sloan

**Affiliations:** 1 Infrastructure and Environment Research Division, School of Engineering, University of Glasgow, Glasgow, United Kingdom; 2 Department of Civil and Environmental Engineering, University of Michigan, Ann Arbor, Michigan, United States of America; 3 The Marine Biological Association of the UK, Plymouth, UK; Purdue University, UNITED STATES

## Abstract

In all but the most sterile environments bacteria will reside in fluid being transported through conduits and some of these will attach and grow as biofilms on the conduit walls. The concentration and diversity of bacteria in the fluid at the point of delivery will be a mix of those when it entered the conduit and those that have become entrained into the flow due to seeding from biofilms. Examples include fluids through conduits such as drinking water pipe networks, endotracheal tubes, catheters and ventilation systems. Here we present two probabilistic models to describe changes in the composition of bulk fluid microbial communities as they are transported through a conduit whilst exposed to biofilm communities. The first (discrete) model simulates absolute numbers of individual cells, whereas the other (continuous) model simulates the relative abundance of taxa in the bulk fluid. The discrete model is founded on a birth-death process whereby the community changes one individual at a time and the numbers of cells in the system can vary. The continuous model is a stochastic differential equation derived from the discrete model and can also accommodate changes in the carrying capacity of the bulk fluid. These models provide a novel *Lagrangian* framework to investigate and predict the dynamics of migrating microbial communities. In this paper we compare the two models, discuss their merits, possible applications and present simulation results in the context of drinking water distribution systems. Our results provide novel insight into the effects of stochastic dynamics on the composition of non-stationary microbial communities that are exposed to biofilms and provides a new avenue for modelling microbial dynamics in systems where fluids are being transported.

## Introduction

A brief review of literature on ecological community assembly yields a plethora of different patterns that often seem contingent on intricate species interaction (e.g. competition, predation) and subtle differences in environmental conditions (e.g. niche). The theoretical models that have been deployed to explain the patterns are almost as diverse as the microbial communities they seek to explain and invoke many different ecological mechanisms. Thus, when confronted with a new system where no comprehensive ecology study has been conducted, wading through the potential models upon which to inform experimental design can be daunting. Vellend (2010) [1], points out that for all their perceived complexity, ecological patterns are influenced by only four broad classes of process: selection, drift, dispersal (or immigration) and speciation. Thus a theoretical framework for a new system should incorporate these processes, or a subset of them, as transparently and simply as possible. Doing this requires a frame of reference. To date models in community ecology, and particularly in microbial community ecology, have primarily resided in an *Eulerian* frame of reference, that is fixed in space with migrants entering and departing via the model domain’s boundaries. In this frame the migrants that are on the move are rarely perceived as a community in their own right, they merely appear as a random sample from some distant location that is unaffected by transit. However, in some environments communities are constantly migrating and influencing their local environment; the Oceanic environment is a prime example [2]. Here a much more natural frame of reference to describe community assembly is *Lagrangian*, where the frame of reference moves with the planktonic community being investigated.

Even if observations on the ecology are taken from fixed locations in space, which is normally easier to engineer, then interpreting the observations ought to be pursued in the light of a conceptual model where communities are in motion [3]. Nowhere is this more so than for the built environment microbiome, which is being recognized increasingly as an interface between humans and microbes that can be potentially engineered to effect changes in health and wellbeing [4]. So, for example, the safety of water delivered at tap or the air that ventilates a hospital ward is of vital importance in disease prevention and the composition of the microbial communities delivered at respective point sources reflects the historical population dynamics within the community as it moved through a complex network interacting with its surroundings. It is not sufficient to simply characterize or model [5] the stationary communities that form, for example as biofilms on the walls of a conduit, to assess risk downstream. Rather, downstream risk should be informed by the biofilm, the bulk fluid overlying the biofilm, and the interactions between the two. The composition of the biofilm phase and planktonic communities in overlying fluid phase often differ [6] with the physical and chemical environments selecting for different microorganisms in the respective phases. If one knows the composition of the community as it sets out on its journey (i.e. initial conditions), from for example, a drinking water plant [4] then using a Lagrangian model that incorporates generic ecological processes of selection, drift and immigration can be used to estimate the likely composition down stream at a point source of interest. Many authors have advocated the formulation of stochastic models in ecology that incorporate, at least, some of the generic processes highlighted by Vellend [1]. Perhaps the most discussed of these in recent years is that of Hubbell [7]. When adopting Hubbels model to describe microbial community assembly, changes to a community occur one individual at a time such that each death is coupled with either an instantaneous local birth (replacement from within the community) or from a source or meta-community (replacement from outside the community). This approach has been used effectively for describing bacterial community assembly in a variety of ecosystems from tree holes to wastewater treatment plants [8–10]. Hubbell’s model assumes microbes can instantaneously migrate and that the total number of microbes remains constant in the local community (i.e. it is saturated).

In the model presented here, we build on Hubbell’s ideas to track the dynamics of bacterial community in a discrete packet of fluid as it moves through the conduit and interacts with the biofilm on the conduit walls. The biofilms on the conduit walls, which may be spatially heterogeneous in composition, can then act as a ‘source’ from which migrants enter the bulk community in the packet of fluid or as a ‘sink’ by allowing individual cells from within the fluid packet of fluid to leave the community and become deposited on the conduit walls. In addition, we relax the neutral assumption by conferring advantage/disadvantages to microbial taxa within the bulk water and biofilm phase and allow total community size to vary within the fluid packet.

We provide discrete and continuous formulations. For example, stochastic birth-death processes have been used for soil and wastewater microbes [11] where the number of individual individuals per unit mass or volume range between 10^7^ − 10^9^. For these community sizes, it is effective to consider the community composition on a continuous scale—i.e. small increments in the relative abundances of microbial taxa rather than modeling community changes on a discrete scale i.e. per individual. In contrast, for systems where low and rare microorganisms are of particular interest (e.g. drinking water systems have range from 10^3^ − 10^5^ cells per ml [6] with rare pathogenic microorganisms are of interest), the discrete formulation of our model may be more applicable.

In this paper we describe the birth/migration-death discrete model and its continuous analogue in the context of a drinking water distribution system where such interactions are considered critical towards understanding the safety of potable water.

## Methods

The discrete model is described in the context of a drinking water distribution system (DWDS), where we consider and simulate the changes in the bacterial community composition of a small packet of bulk water as it travels through a drinking water distribution system. The packet of bulk water is exposed to biofilms on the pipe walls as it travels through and the bulk water community is subject to migration of individuals from the biofilm community.

The model is founded on a stochastic birth-death process with migration from a ‘source’ or ‘meta’ community (in this case the pipe wall biofilm community). This process is utilized in previous work [11, 12] and since it provides the foundation of this model we describe it here using the following notation. We let:

- *N* denote the total number of individuals in the community at the start (for the discrete model).
- *r* denote the total number of taxa in the source and initial communities including the unfilled capacity taxon.
- **p** denote the vector (length *r*) of relative abundances in the biofilm community.
- **f** denote the vector (length *r*) of relative abundances in the initial bulk water community.
- *α* denote a vector (length *r*) of selective advantages.
- *β* denote a vector (length *r*) of selective disadvantages.
- **x** ^*t*^ denote the vector (length *r*) of relative abundances at time *t* in the water community. Note that this changes as events occur over time.
- *m* denote the migration rate; i.e. the probability that given a death event, the individual is replaced via a migration event rather than a local birth event.

### Migration, Births and Deaths

The water packet community composition changes via individual death events coupled with either a local community birth/replication event or a migration event. The community composition changes one individual at a time, so for larger populations more events are required to bring about the same changes in community composition with respect to relative abundances. We would expect the number of events in a unit of time to be proportional to the number of individuals in the sample. Mapping time in events to real time requires system specific scaling (see discussion).

At each event the individual ‘selected’ to die and the individual selected to replicate or migrate are chosen at random according to the proportions in which they are present in the bulk water and in the biofilm. In a model where there are no selective pressures on any taxa, the death, local birth and migration probabilities for each taxa are exactly equivalent to its proportion in the water or biofilm.

### Selective Advantages and Disadvantages

To model selective pressures, we introduce two vectors of weights; *α* and *β* of length *r*. The probability an advantaged taxa *i* is selected to replicate is the current relative abundance upweighted by *α*_*i*_ and the probability a disadvantaged taxa *j* is selected to die is the current relative abundance upweighted by *β*_*j*_. Hence the taxa local birth and death probabilities at time *t* (denoted **b** ^*t*^ and **d** ^*t*^) are functions of weighted relative abundances (equations 1 and 2).

$$\begin{array}{c} b_{i}^{t}=\frac{\left(1+\alpha_{i}\right)x_{i}^{t}}{1+\sum_{j=1}^{r}\;\alpha_{j}x_{j}^{t}} \end{array}$$ (1)

$$\begin{array}{c} d_{i}^{t}=\frac{\left(1+\beta_{i}\right)x_{i}^{t}}{1+\sum_{j=1}^{r}\;\beta_{j}x_{j}^{t}} \end{array}$$ (2)

We assume all biofilm taxa are equally likely to migrate into the bulk water and, once they have migrated and become part of the local community they become subject to selective pressures. For example a new biofilm taxa might migrate into the local community and thus be present with relative abundance 1/*N* in the local community. With a strong disadvantage this individual might be highly likely to be the individual chosen for the next death event.

### Carrying Capacity of Media

Changes in the carrying capacity of the water (or other media) are modeled via the introduction of the carrying capacity buffer. It behaves exactly as any other taxon in the system and allows the number of individuals in the water packet to be increased up to a pre-defined maximum. For example the community size may grow by 20% over time, reflecting changes in environmental conditions such as falling disinfectant levels in drinking water distribution systems. An increase in overall community size equates to a reduction of the carrying capacity buffer. When there is no carrying capacity buffer remaining, the water is at maximum capacity.

### Sloughing

The model incorporates the continuous removal of individual cells from the biofilm community using single migration events as part of the birth-death process. This accounts for cells dispersed into the bulk water community by shedding of daughter cells. However, the biofilm community may also be dispersed into the bulk water by detachment of biofilm aggregates by shearing. We term these events ‘biofilm sloughing events’ (rather than biofilm migration events) and in these instances, there is a large influx of individuals simultaneously into the bulk community. Consequently, when biofilm on the water distribution pipe wall sloughs into the bulk water, the bacterial community in the water packet can change significantly.

To model the possibility of a biofilm event we introduce an extra parameter (*s*), the sloughing rate. Each event involves a death coupled with either a local birth, birth from source community or a sloughing event with probabilities (1-*s*-*m*), *m* and *s* respectively. In the case the event is a sloughing event we introduce a large number of individuals (*N*_*s*_) to the water packet with taxa frequencies reflecting the biofilm community composition. The influx of new individuals to the system can be handled in various ways and adapted to the context. For drinking water distribution systems, the disinfectant present in the water is likely to kill a large proportion of the sloughed individuals. The resulting increase in biomass in the system may increase the carrying capacity of the water. To reflect this in the modeling strategy we allow a proportion of the biofilm individuals to die instantly (*p*_*die*_), resulting in a corresponding increase in the carrying capacity buffer.

In our simulations we set

- *s* = 10^−6^
- *m* = 0.04
- *N*_*s*_ = 10^3^
- *p*_*die*_ = 0.5

### The Continuous Model

Where communities are large, say of order 10^9^ individuals per unit mass or volume, we can take a continuous limit and represent the discrete process with many taxa as a multidimensional diffusion process. Doing this involves letting the population size N tend to infinity whilst some parameters remain of order 1/N ([11] supplementary material). The resulting stochastic differential equation is a diffusion equation of the form

$$\begin{array}{c} dX=Mdt+\sigma \sqrt{V}dW_{t} \end{array}$$ (3)

where *X*(*t*) is a vector of relative taxa abundances in the community at time *t*, *W*_*t*_ is a standard multidimensional Brownian motion, M is a vector drift term and *V* is a variance matrix. The latter two are given below:

$$\begin{array}{ccc} M_{i} & = & m^{*}\left(p_{i}-X_{i}\right)+\alpha_{i}^{*}X_{i}-\left(\alpha^{*}\cdot X\right)X_{i}\\ \alpha^{*} & = & N(\alpha -\beta ) \end{array}$$ (4)

where **p** is the vector of relative abundances in the biofilm or source community as in the discrete model, parameters m* and α* are scaled versions of migration rate and weightings in the discrete model, so that

$$\begin{array}{ccc} m & = & \frac{m^{*}}{N}\\ \alpha -\beta & = & \frac{\alpha^{*}}{N} \end{array}$$ (5)

If a single unit of time is equivalent to *N* ^2^ single events then *σ* = 1. The variance matrix *V* has components

$$\begin{array}{ccc} V_{ii} & = & 2X_{i}\left(1-X_{i}\right)\\ V_{ij} & = & -2X_{i}X_{j}\;\;i\neq j \end{array}$$ (6)

Since the relative abundances must sum to unity, the variance matrix is singular of rank *r* − 1, where *r* is the total number of taxa including the unfilled capacity taxon. The benefit of the continuous model is that simulations may be run without reference to the overall population size N so that such simulations are more efficient.

The discrete and continuous models (with matched parameters) are equivalent when the total community size is large and advantage terms *α* − *β* and migration rate *m* are of order $\frac{1}{N}$. This is demonstrated in our results.

### Source Community Heterogeneity

In the results presented here, we assume the source/biofilm community composition remains constant throughout the system and over time but there is no need to do so. This serves as an effective modeling assumption in the case of drinking water distribution systems where the biofilms are well established and relatively stable. Further although there may be considerable heterogeneity in the pipe biofilm communities from meter to meter of pipe, over a large stretch of pipe we may reasonably assume that the water packet has been exposed to the same biofilm ‘source’ community. For other applications, this assumption can be relaxed by drawing the biofilm or ‘source’ relative abundances at each event from a probability distribution.

## Results

We use water flowing through drinking water pipes by way of an example to demonstrate the value of our modeling approach. In the first instance we considered a community made up from 3 distinct taxa.

One taxon represents individuals already present in the bulk water (referred to as the bulk water taxon), another represents individuals present on the biofilm (referred to as the biofilm taxon) and a third, which might not normally be considered a taxon, represents the unfilled capacity in the bulk water (referred to as the carrying capacity buffer). Though the approach of lumping all taxa within the three initial communities into a single taxon may represent an over-simplification of real microbial communities, it provides us with a good starting point and is also consistent with the neutral assumption where all species which comprise a taxon may be considered equivalent. The carrying capacity buffer is viewed as the space (a count for the discrete model or proportion for the continuous model) within the community which could be occupied either due to migration of individuals from the biofilm into the bulk water or growth of individuals from within the bulk water community. By including a carrying capacity buffer, we are relaxing the limitation of saturated community requirements of a neutral theory, without violating other assumptions of the neutral theory (i.e. equivalence between taxa). Specifically, the carrying capacity buffer, whilst not strictly a taxon in the community, modeled as one in order to increase or decrease the carrying capacity of the bulk water. Under neutrality, by considering the space like any of the other taxa in the system, it is equally likely to ‘die’ (i.e. filled) and be ‘born’ (i.e. created).

We have also assumed that just before the packet of water enters the conduit, there is no shared membership between the bulk water and the biofilm communities. This assumption simplies a drinking water distribution system, yet is supported by literature. [6, 13–15] where biofilm communities have been shown to be distinct to bulk water communities. Using the notation described in the methods section, we use the parameters below. For vector parameters of length three, the first element corresponds to the carrying capacity buffer, the second element corresponds to the bulk water taxon and the final element corresponds to the biofilm taxon. In all simulations we set

- **f** = (0.2, 0.8, 0) where 0.2 is the relative abundance of the carrying capacity buffer, 0.8 is the relative abundance of the bulk water taxon, and 0 indicates the absence of biofilm taxon in the bulk water initially.
- **p** = (0, 0, 1), indicating only the biofilm taxon is present on the pipe walls.
- *m* = 0.04, this migration rate is based on previous estimates [11].
- *N* = 10^3^, Note that in the continuous model it is not necessary to define *N*.

We keep these parameters fixed and vary the advantage and disadvantage terms *α* and *β* between zero and one for the discrete model. α* denotes the net selection parameter in the continuous model. It is defined by (advantage-disadvantage)×*N* (see methods) and varies from -100 (disadvantage) to +100 (advantage) in our simulations. We refer to selective advantages and disadvantages collectively as selective pressures in any given instance. Notably we see that seemingly very small selective pressures have large effects in the community dynamics.

**Fig 1:**
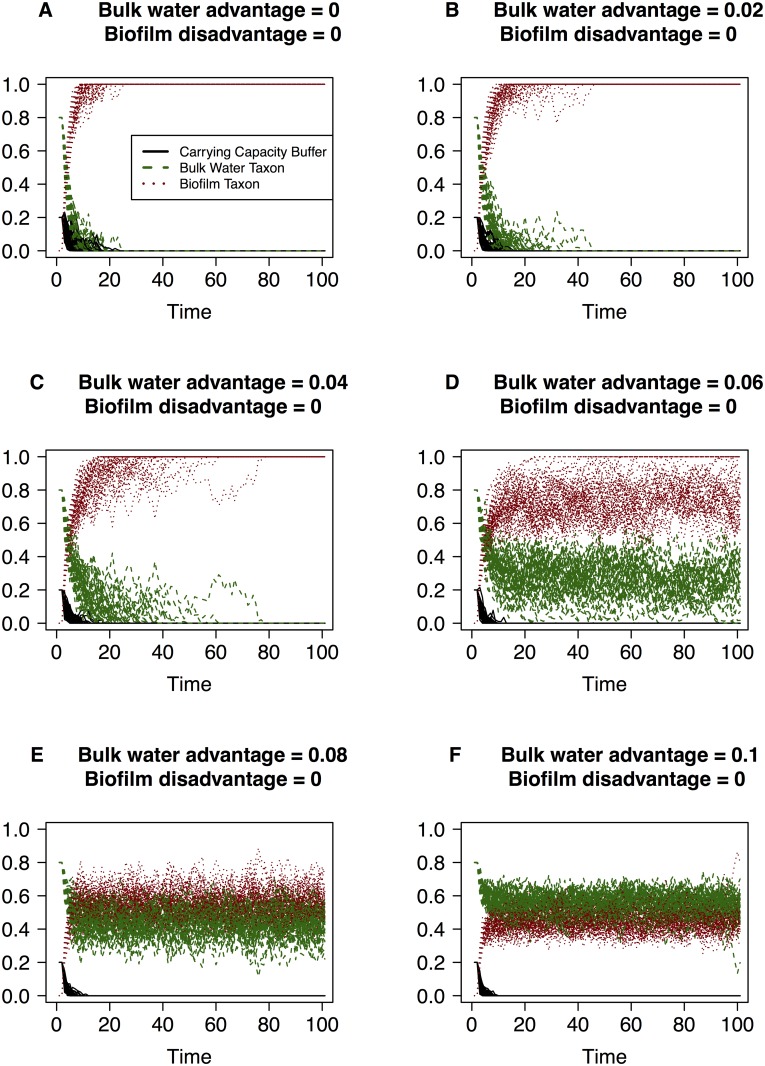
Simulations of the changes in community composition according to the discrete model when an increasing selective advantage is given to the bulk water taxon. The vertical axis is the taxon relative abundance and the values of each taxon (i.e. the black, green and red lines) sum to one at each point along the horizontal axis. Magnitude of the advantage terms are labeled in each graph (ranging from zero to 0.1) and time (the horizontal axis) is measured in units of 10^4^ events.

**Fig 2:**
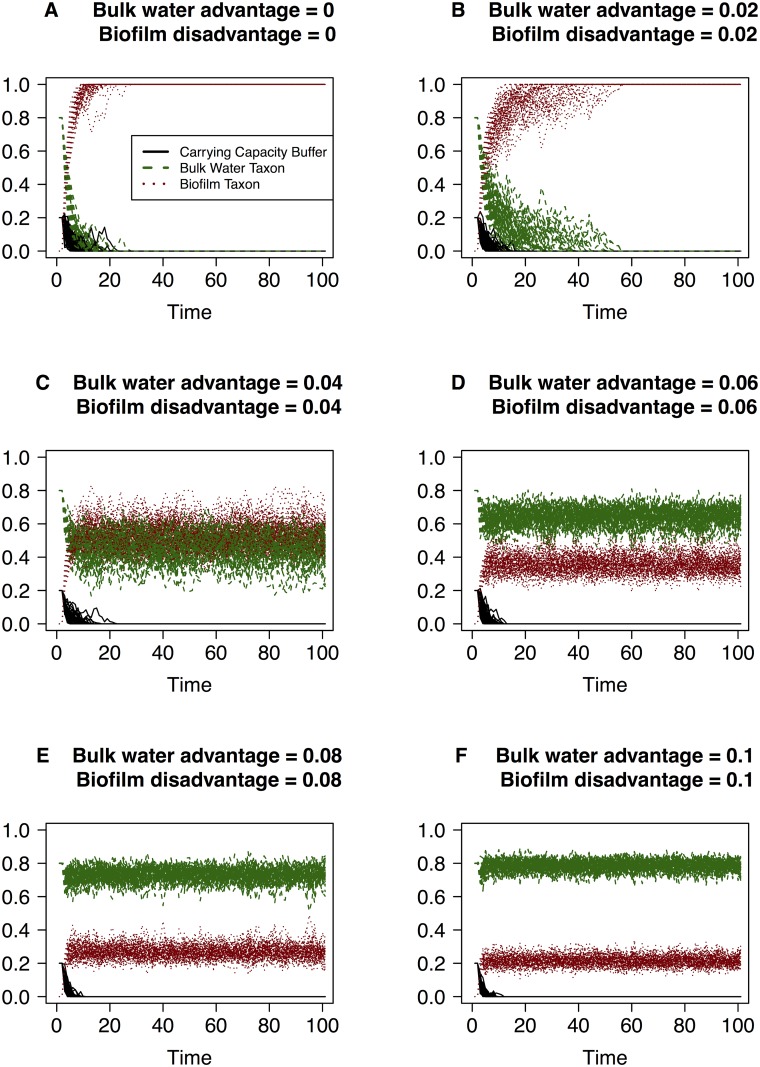
Simulations of the changes in community composition according to the discrete model when a selective advantage is given to the bulk water taxon and a selective disadvantage of the same magnitude is given to biofilm taxon. The vertical axis is the taxon relative abundance and the values of each taxon (i.e. the black, green and red lines) sum to one at each point along the horizontal axis. Magnitude of the advantage and disadvantage terms are labeled in each graph, ranging from zero to 0.1 and time (the horizontal axis) is measured in units of 10^4^ events.

**Fig 3:**
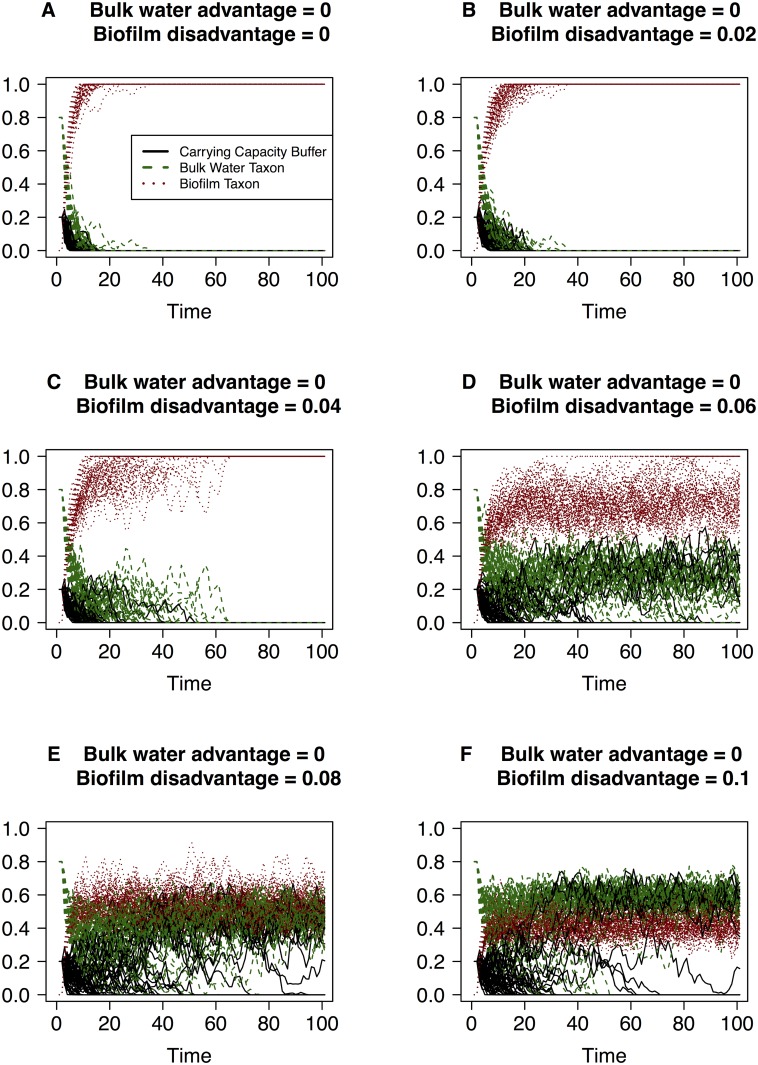
Simulations of the changes in community composition according to the discrete model when a selective disadvantage is given to the biofilm taxon. The vertical axis is the taxon relative abundance and the values of each taxon (i.e. the black, green and red lines) sum to one at each point along the horizontal axis. Magnitude of the disadvantage terms are labeled in each graph ranging from zero to 0.1 and time (the horizontal axis) is measured in units of 10^4^ events.

We consider the relative abundances of the three taxa over time under the following three scenarios:

1. Selective advantage (*α*) to the bulk water taxon (Fig. 1 and Supplementary S1 Fig. for the discrete and continuous models respectively).
2. Equal selective advantage (*α*) to the bulk water taxon and disadvantage (*β*) to the biofilm taxon, i.e. *α*_*bulk*_ = *β*_*biofilm*_. (Fig. 2 and Supplementary S2 Fig. for the discrete and continuous models respectively).
3. Selective disadvantage (*β*) to the biofilm taxon (Fig. 3 and Supplementary S3 Fig. for the discrete and continuous models respectively).

In each of these scenarios, the carrying capacity buffer has neither an advantage nor disadvantage (i.e. is purely neutral).

In the discrete model, magnitude of the advantage and disadvantage terms range from zero (i.e. the neutral model) up to 0.1 with five increments of 0.02. We focus on a small range of advantage/disadvantage parameters to allow for a clear visualization of the effects of varying these parameters.

When there is a selective advantage to the initial bulk water taxon and disadvantage to the biofilm taxon (i.e. scenario 2 above), the magnitude of the terms are kept the same. The equivalent parameter values in the continuous model are α* = 0, ±20, ±40, ±60, ±80, ±100. Positive terms correspond to advantages and negative terms to disadvantages. For each of the aforementioned conditions, we ran 50 simulations from the same initial starting values and recorded the relative abundances of each of the three groups at each step of the simulation over time (Figs. 1, 2 and 3 for the discrete model and Supplementary S1, S2, S3 Figs. for the continuous model). To summarize and compare runs, we consider the mean and variance of the relative abundance of each group within each simulation once the system has reached stability and where appropriate we consider the average time taken for one group to die out of the system within each simulation or the proportion of times the group dies out across the 50 simulations (S1 Table, S2 Table, S3 Table).

The simulations reveal that in the mock system with three taxa or groups, sufficient migration of biofilm taxon into the bulk water will result in it dominating the bulk water community under strictly neutral conditions (Figs. 1A, 2A, 3A). This dominance of the bulk water phase by the biofilm taxon is observed even under conditions where the bulk water taxon has a small advantage (Figs. 1B-1D) or the biofilm taxon has a low level disadvantage (Fig. 3B-3D). This feature arises in the simulations because the abundance of the biofilm taxon in the bulk water community is replenished due to both growth in the bulk community as well as by migration from the conduit walls. In contrast, the replacement of a bulk water taxon individual only occurs due to the growth within the system. It takes a significant advantage attributed to the bulk water taxon and concurrent significant disadvantage attributed to the biofilm taxon to ensure that the bulk water taxon dominates the bacterial community in the packet of water (Figs. 2D-2F). These simulations provide us with critical insight into the conditions that must be present to reflect these conditions in real systems. To ensure that the bulk water taxon is not entirely replaced by the biofilm taxon, the bulk water and biofilm taxon must experience significantly different selective pressures (Fig. 3D). In the built environment, this may arise in a myriad of ways, with the likely biggest factor being the physiological differences under relevant environmental conditions (e.g. substrate composition and availability, disinfectant stress, conduit wall properties) between biofilm growth mode and planktonic state bacteria.

In this model we assume no overlap between the bulk water and biofilm communities. It is likely, that such assumption must be relaxed when applying this model to experimental and/or field data. In particular, under steady state conditions the biofilm will represent a mix of the biofilm and bulk water taxon (subject to selective pressures in the biofilm environment) and both taxa would migrate into the bulk water as a function of the migration rate and their relative abundance on the biofilm.

Another interesting aspect of the model is its ability to simulate changes in the community size (not just community membership) in the packet of fluid. Fig. 3 clearly illustrates that certain parameter conditions can lead to instability within a system i.e. the system does not settle about the same seemingly steady state distribution after *N* ^2^ events as observed in Figs. 1 and 2. Specifically, we see that under conditions where biofilm taxon has a significant disadvantage and the bulk water taxon has no advantage/disadvantage (Fig. 3F), the carrying capacity buffer fluctuates significantly. This feature arises from the fact that a death of bulk water taxon individual is equally likely to be replaced by another bulk water taxon or a carrying capacity buffer, and the disadvantage attributed to the biofilm taxon is high enough that it is less able to participate in a replacement event despite the high migration rate. When only the biofilm taxon is disadvantaged the behaviour of the system depends on the magnitude of the disadvantage. When it is less than or equal to the migration rate the biofilm taxon still completely dominates the system (Fig. 3 A-C). When the disadvantage is greater than the migration rate (Fig. 3 D-F) both the biofilm taxon and bulk water taxon remain in the system over this time course. The only difference between them is their initial conditions and it thus follows that in the long run they become indistinguishable. The inability to distinguish between biofilm and bulk water taxa is relevant to taxonomic groups which share the same selective pressures and migration rates. Within these groups, stochasticity in the system may mean that some taxa die out and others become dominant simply by chance.

In our simulations we kept the migration rate fixed to investigate the effect of changing the selective pressures. With neutrality we see that the carrying capacity buffer and the initial bulk water taxa are eliminated from the system after approximately 10^5^ events, with the bulk water taxa taking slightly longer reflecting the difference in initial conditions. The time taken for elimination of the bulk water taxa from the system correlates negatively with the migration rate such that an increase in migration rate of the biofilm taxon reduces the time taken for the bulk water taxa to be to be eliminated.

Eventually in all of our simulations where the bulk taxon has an equal and opposite advantage to biofilms disadvantage, the carrying capacity buffer is eliminated so that the other taxa run at full capacity in the fluid. Further, there is no indication in simulation results that the bulk taxon disappears from the system using larger disadvantage pressures. From the theory when just two taxa are present and similarly for when there are more than two, there is an absorbing boundary when the only taxon which can migrate in reaches full occupancy and so has relative abundance of 1. Theory (S1 Methods) shows there is a non-zero probability of reaching this boundary in finite time. Furthermore, we have estimated the expected time to absorption (i.e. the migrating taxa reaches dominance) in the case of a simple system with two taxa, one migrating and the other present initially (Supplementary S4 Fig.). The expected times to absorption are enormous when the selective pressure is large, thus explaining our observed simulation results.

**Fig 4:**
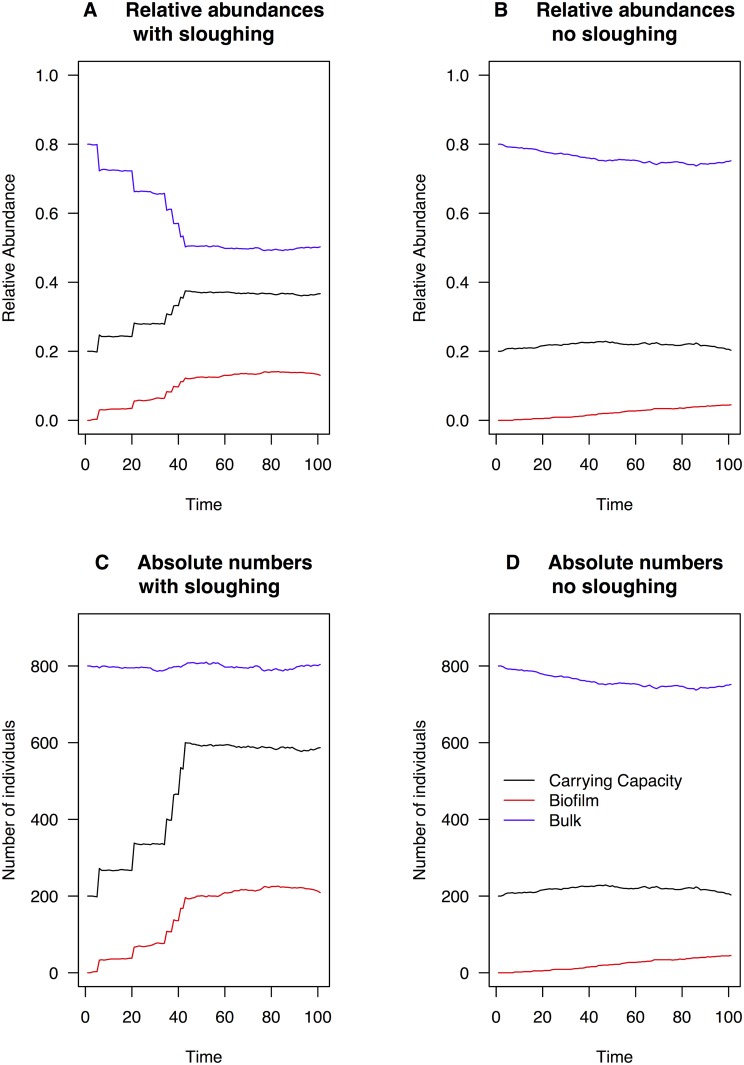
Simulations for the discrete model with and without sloughing. Figures 4A and 4C illustrate instances with sloughing and 4B and 4D without sloughing. In this example intially *N* = 1000 with the number of individuals migrating into the water packet at a single sloughing event equal to 1000. We see an increase in the carrying capacity of the water introduced by the increased biomass and an increase in the number and proportion of biofilm introduced individuals. Sloughing events are characterised by larger jumps in both the relative abundance and the absolute numbers.

To further investigate the impact that biofilm sloughing has on the community composition we also ran the discrete model with sloughing (see methods) over a shorter period of time to mimic the timescale in a water distribution system (Fig. 4). The continuous model cannot naturally incorporate an influx of a large number of individuals at an instant. When there a biofilm sloughing event, the total population size in the water packet increases. Thus there is an increase in absolute numbers of bacterial cells in the water. We assume that a certain proportion of the sloughed individuals die instantly due to selective pressure (for example due to disinfectant) and increase the carrying capacity to reflect the increase the dead biomass provides to the existing community. These are parameters in the model (see methods) which can be adjusted to reflect the environment.

In our simulations (Fig. 4) we use the following parameters:

- Starting relative abundances of the water packet **f** = (0.2, 0.8, 0) where 0.2 is the relative abundance of the carrying capacity buffer, 0.8 is the relative abundance of the bulk water taxon, and 0 indicates the absence of biofilm taxon in the bulk water initially.
- Source community composition **p** = (0, 0, 1), indicating only the biofilm taxon is present on the pipe walls.
- Migration rate *m* = 0.04
- Sloughing event rate *s* = 10^−6^
- Local community birth rate = 1 − *m* − *s*
- Original number of cells in the water packet *N* = 10^3^
- Number of cells introduced to the water packet at a sloughing event *N*_*s*_ = 10^3^
- Proportion of cells introduced to the water packet by the sloughing event which die instantly *p*_*die*_ = 0.5

To compare the behaviour of the continuous model and discrete model without sloughing, we ran the continuous model with matching parameters for the same period of time (Supplementary S1, S2 and S3 Figs. analogous to Figs. 1, 2 and 3 in the main text). The changes in relative abundance are typically larger in the continuous model (relative to 1/*N* in the discrete model) and this reflects the fact that in this model it is possible to adjust the increment in relative abundances at each step. This is comparable to multiple events occuring simultaneously in the discrete model (i.e. more individuals die and are born) and makes the continuous model more efficient to run and more suitable for simulating community changes over time when the total community size is large. The simulations are comparable (S1 Table, S2 Table and S3 Table) even when the magnitude of the advantage term extends beyond the range for which the approximation is expected to hold (see methods).

## Discussion

The discrete model provides an intuitive way to model the abundances of different bacterial taxa in a system as it moves through space over time. It can readily incorporate selective pressures, varying carrying capacity and an influx of a large number of individuals at a single time point. The ability to handle biofilm-sloughing events distinguishes it from the continuous model, which can only adjust its carrying capacity up to a predefined maximum as a proportion of the individuals already present in the system. This makes the discrete model effective for assessing bacterial abundance levels when the absolute abundances (rather than relative abundances) are of importance.

Time in our model is measured in discrete birth-death events. How this relates to real time depends on the population size. In small population you might expect to wait a relatively long time between successive births and deaths in comparison to a very large population where births and deaths would be occurring somewhere in the community almost continuously. So the real time between events speeds up with population size. However, the number of events required to effect a significant change in the relative abundance is also affected (non-linearly) by population size. So, for example, the expected number of events required for every one of the original *N* taxa to have been replaced once is *N* ^2^. Thus simulating for long enough to reach some dynamic equilibrium in a large community (> 10^5^) organisms is cumbersome with the discrete model. Although in cases such as drinking water systems, whether microbial communities communities would ever reside in the pipes long enough to reach such an equilibrium is a mute point. The actual time between events will be situation specific and will only be determined by calibration of either the discrete or continous model.

Sloughing events can be incorporated into the discrete model easily with extra parameters. They govern the rate at which sloughing events occur, the number of individuals sloughed in, their survival rate and the subsequent increase in the carrying capacity of the water (e.g. addition of growth substrates due to microbial death and decay). Hence there is potential here for modeling more complex behaviours where, for example, the number of individuals sloughed is a function of hydraulic regime [16] or carry capacity is a function of the dissolved organics [17]. In contrast, the continuous model is of importance when the population size is large (say order 10^9^) and/or when long term behaviour of a system is of interest. A further benefit to using this model is that approximate likelihood methods can be implemented for parameter calibration [11].

In the case of drinking water distribution systems, the total number of events incurred as the bulk water passes through the distribution network to its endpoint is likely to be very much fewer than the number presented in our simulation results. The length/duration (i.e. number of events) of our simulations were chosen to demonstrate stochastic behavior of the system. We note that for other systems long-term community behaviour is of high importance. Our modeling framework could be adapted to model these communities and may provide valuable information regarding protocols for seeding and fixing a dispersing bacterial community into the intended environment successfully in the long term. For example, our modelling approach could be used to inform strategies for bioaugmentation in wastewater [18] or contaminated land remediation [19] scenarios, or even to inform strategies for microbial transplants [20].

In the simulations presented here, we assumed the biofilm community is homoegeneous through the network and over time. In reality, mixed community biofilms are highly heterogeneous (in physical structure and variance in community composition over spatial scales [21]) and thus it is challenging to sample them in a representative manner [6]. As a result, model predictions relating to change in biofilm community would be very difficult to validate experimentally. Rather, than model the difficult to verify heterogeneity in biofilm composition we simplify our model by assuming that the migrating bulk water community is exposed to a biofilm community homogeneous over large spatial scales. With this approach, we can infer the overall biofilm community by quantifying the difference in bulk water community between initial and final conditions (one end of a pipe to the other). In drinking water distribution systems, the primary interest lies in the bulk community since it is microbial communities in bulk water being consumed by the customer. We acknowledge that in some circumstances modeling the biofilm communities may be of interest (e.g. biofilms on intestinal walls) and in such situations, our model provides a framework that can be modified appropriately to incorporate features of specific systems. This might include modelling changes in the over all size of the biofilm community and/or changes in migration and selection parameters to reflect the biofilm formation process more accurately.

In the simulations shown, parameters are kept constant throughout each simulation run. However this need not be the case; parameters can be varied to reflect changes in other variables. For example, decay in disinfectant residual can be modeled by increasing the carrying capacity of the packet of fluid or by decreasing disadvantage terms to taxa susceptible to disinfection. Indeed, if there are measurable variables known to effect fitness of a taxonomic group this information could be used to aid parameter estimation. We note that for the continuous model it is not possible to estimate or set N = total number of individuals in the system since this parameter is coupled with time. This can be advantageous in scenarios when the sample size is large (e.g. fluvial networks [22]).

The models presented here both assume that the migration parameter is homogeneous for all taxa comprising the biofilm community (in our case grouped to a single ‘biofilm taxon’). We acknowledge this assumption may not reflect differences in motility or capability to migrate into the bulk water. These differences can be accounted for by defining an ‘effective’ biofilm community which is weighted accordingly. After applying this weighting, all taxa can be assumed to migrate with equal probability.

With these simulations we can address issues such as the role of selective pressures and migration rates on microbial dynamics in a large variety of systems. For example, the model can inform the application/engineering of selective pressures (guided by parameter choice) to minimize the risk of contamination of water systems from detrimental microorganisms (e.g. pathogens). Further, we can model a system that does not reach steady state by running it with a shorter time horizon. The same simulation methods can be applied to assess pathogen contamination risk. Given a set of simulations we can obtain a Monte Carlo estimate for expected pathogen abundance and estimate contamination risk using the proportion of runs the pathogen abundance exceeds the danger threshold. Alternatively, the continuous model could be used to derive functions for the expected risk exactly.

Similarly, we can apply these models to systems where taxa are purposefully inoculated into a system with the aim of becoming fixed into the system despite the dynamics of microbes that are native to system itself (e.g. probiotics). Seeding success rate is the expected proportion of times the seeded taxa remains in the system for a period of time above a certain relative abundance. The model can provide insight into the frequency of inoculation/seeding and/or the selective pressures necessary for the seeded taxon to fix in the system rather than die out. By increasing the selective advantage of the initial bulk water taxon (or the seeded taxon in this case) we see it first taking longer to be eliminated from the system, then, as the advantage term increases beyond the migration rate, we see that it stays in the community at a relative abundance that increases with the magnitude of the selective advantage (Fig. 1). More generally if a taxon was seeded into a population and the selective advantage for that seeded taxa exceeded the growth and migration rates, (given no other selection) the seeded taxon is highly likely to remain in the system in the long run.

The approach we describe here can also be used to model the converse; bacterial community assembly for biofilms. To do this, we would consider the bulk water community as the migrating community and track the formation and stability of the biofilm community. In these circumstances it would be appropriate to model the long-term behaviour of the system. These communities although very diverse are typically established over many years. Our work shows how stochasticty inherent in community dynamics can result in taxa being eliminated from a system or fixing by chance.

## Supporting Information

S1 Fig Simulations from the continuous model with a selective advantage given to the bulk water taxon. The advantage term α* for the bulk water taxon ranges from zero to 100 (as labelled). This allows for easy comparison with Fig. 1 of the main paper since the paramaters are equivalent in the two frameworks.
(TIFF)

S2 Fig Simulations from the continuous model with a selective advantage given to the bulk water taxon and a selective disadvantage of equal magnitude to the biofilm taxon. The advantage/disadvantage terms (displayed in the titles) range from zero to ± 100 (positive for advantage to bulkwater taxon and negative for disadvantage to biofilm taxon). This allows for easy comparison with Fig. 2 of the main paper since the paramaters are equivalent in the two frameworks.
(TIFF)

S3 Fig Simulations from the continuous model with a selective disadvantage given to the biofilm taxon. The disadvantage parameter for the biofilm taxon (displayed in the titles) ranges from zero to -100). This allows for easy comparison with Fig. 3 of the main paper since the paramaters are equivalent in the two frameworks.
(TIFF)

S4 Fig Expected times to absorption for a 2-taxa system. In the 2-taxa-system, the function *T*(*x*, *b*) is as defined in Supplementary S1 Methods for a range of advantage terms, taking initial relative abundance b = 0.8. Time to fixation is 100× area under the curve, in each graph. The advantage term α* ranges from zero to 100.
(PDF)

S1 Table A comparison of the average mean and variability (after reaching seemingly stable state) for each taxon relative abundance when a selective advantage is given to the bulk taxon and no advantage or disadvantage is given to the biofilm or carrying capacity buffer. The average was computed in the same way for both the discrete and continuous simulations. For each of the 50 runs, once the system had settled to a seemingly steady state, we computed the mean relative abundance level and the variability about this mean for each of the taxa. The averages presented here are averages of these quantities estimated from 50 independent simulations when selective advantage (*α*) is given to the bulk taxon and no advantage or disadvantage is given to the biofilm or the carrying capacity buffer.
(PDF)

S2 Table A comparison of the average mean and variability (after reaching seemingly stable state) for each taxon relative abundance when when selective advantage (*α*) is given to the bulk taxon and selective disadvantage of the same magnitude is given to the biofilm taxon. The average was computed in the same way for both the discrete and continuous simulations. For each of the 50 runs, once the system had settled to a seemingly steady state, we computed the mean relative abundance level and the variability about this mean for each of the taxa. The averages presented here are averages of these quantities estimated from 50 independent simulations when selective advantage (*α*) is given to the bulk taxon and selective disadvantage of the same magnitude is given to the biofilm taxon. The carrying capacity buffer is not given a selective advantage or disadvantage.
(PDF)

S3 Table A comparison of the average mean and variability (after reaching seemingly stable state) for each taxon relative abundance when a selective disadvantage is given to the biofilm taxon. The average was computed in the same way for both the discrete and continuous simulations. For each of the 50 runs, once the system had settled to a seemingly steady state, we computed the mean relative abundance level and the variability about this mean for each of the taxa. The averages presented here are averages of these quantities estimated from 50 independent simulations when a selective disadvantage (*β*) is given to the biofilm taxon and no advantage or disadvantage is given to the bulk or carrying capacity buffer.
(PDF)

S1 Methods Theoretical Expected Times to Absorption. (PDF)
