# Supplementary figures and images for: Probabilistic Models to Describe the Dynamics of Migrating Microbial Communities
Source: PLoS One. 2015 Mar 24;10(3):e0117221. doi: 10.1371/journal.pone.0117221 (PMC4372544; doi:10.1371/journal.pone.0117221)

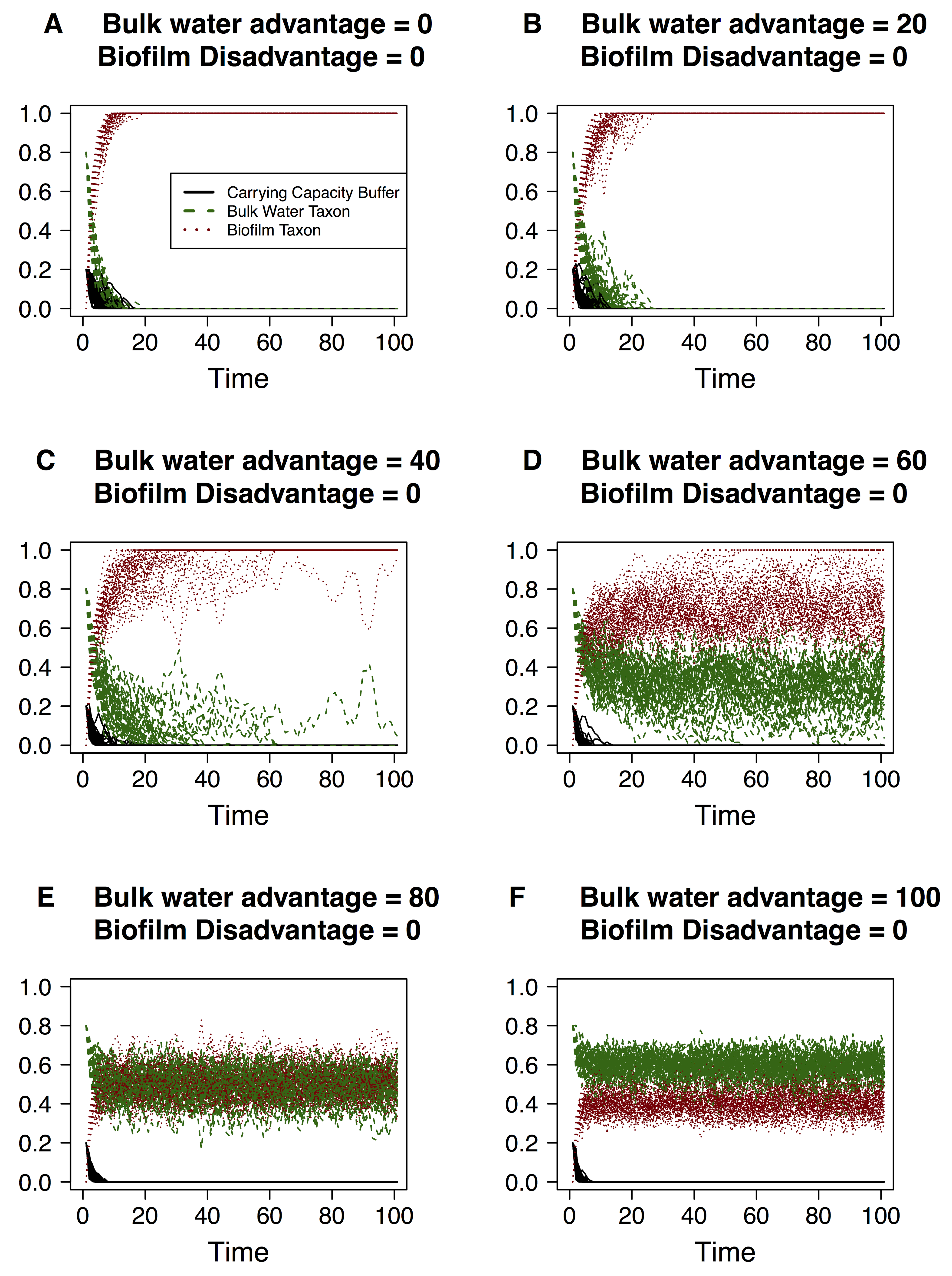

Supplement: S1 Fig — The advantage term α* for the bulk water taxon ranges from zero to 100 (as labelled). This allows for easy comparison with Fig. 1 of the main paper since the paramaters are equivalent in the two frameworks. (TIFF) [file pone.0117221.s001.tiff]

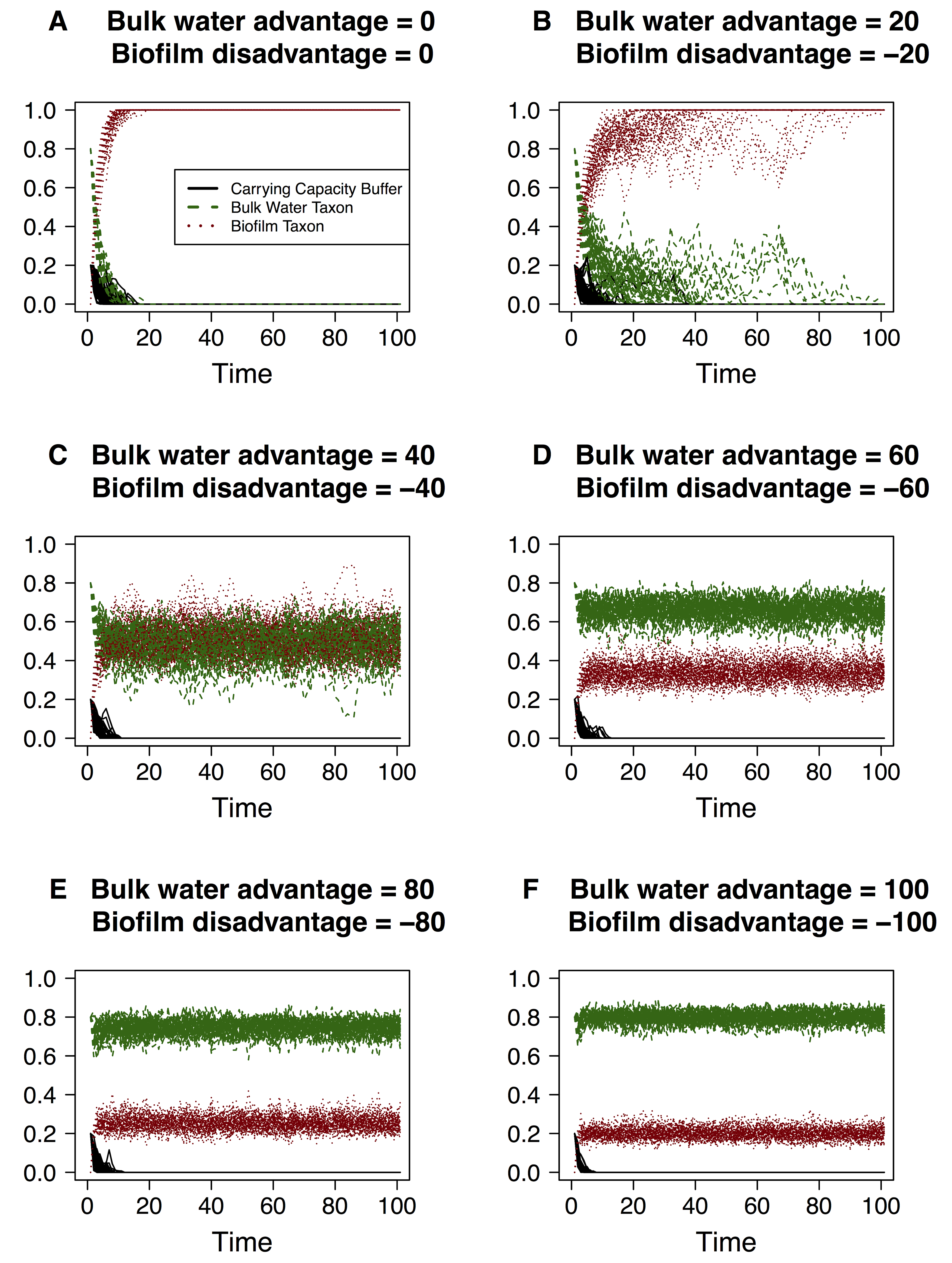

Supplement: S2 Fig — The advantage/disadvantage terms (displayed in the titles) range from zero to ± 100 (positive for advantage to bulkwater taxon and negative for disadvantage to biofilm taxon). This allows for easy comparison with Fig. 2 of the main paper since the paramaters are equivalent in the two frameworks. (TIFF) [file pone.0117221.s002.tiff]

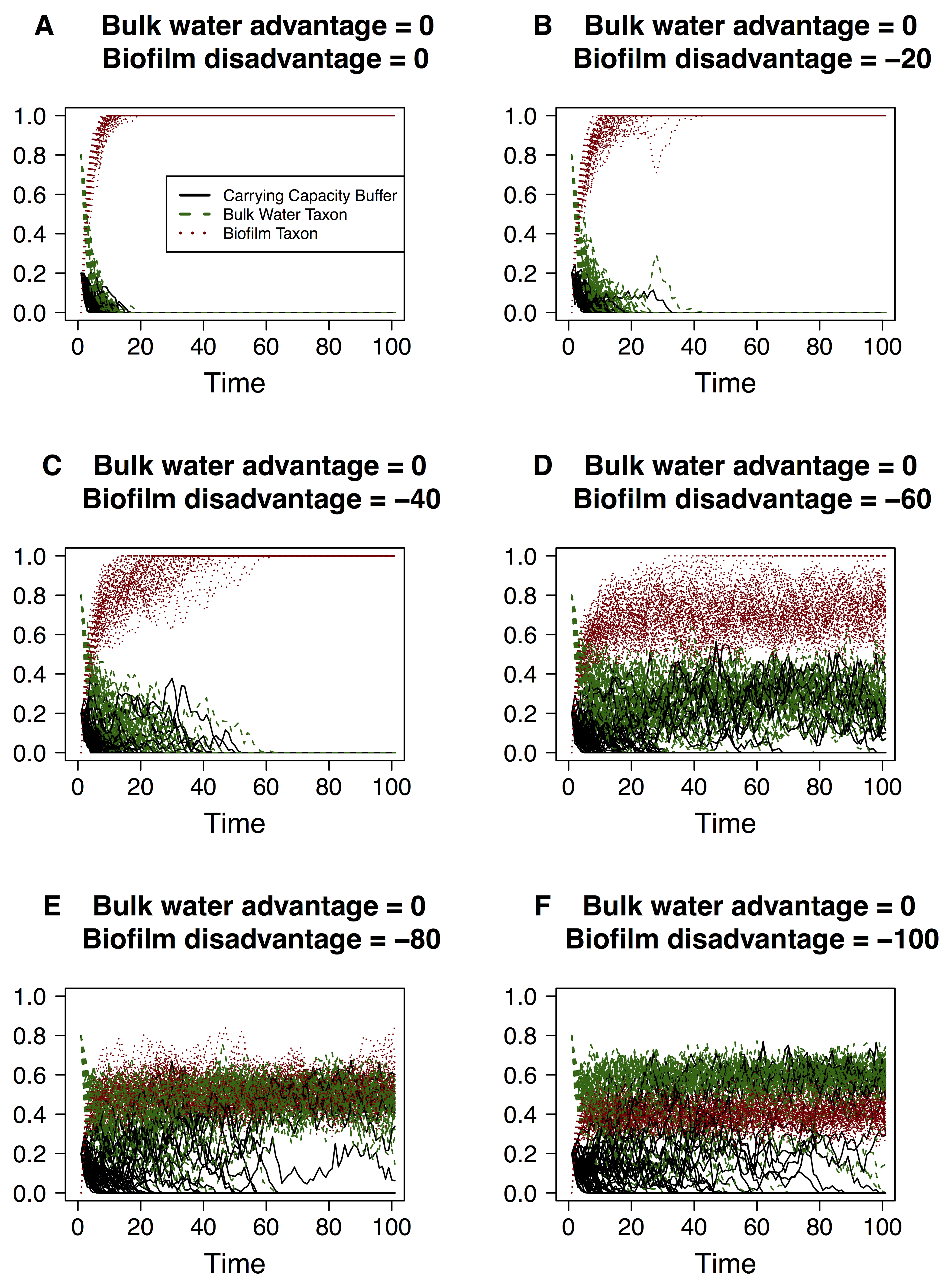

Supplement: S3 Fig — The disadvantage parameter for the biofilm taxon (displayed in the titles) ranges from zero to -100). This allows for easy comparison with Fig. 3 of the main paper since the paramaters are equivalent in the two frameworks. (TIFF) [file pone.0117221.s003.tiff]
