# Supplementary material for: Probabilistic Models to Describe the Dynamics of Migrating Microbial Communities
Source: PLoS One. 2015 Mar 24;10(3):e0117221. doi: 10.1371/journal.pone.0117221 (PMC4372544; doi:10.1371/journal.pone.0117221)

Probabilistic models to describe the dynamics of migrating microbial communities

Joanna L Schroeder, Mary Lunn, Ameet J Pinto, Lutgarde Raskin, William T Sloan

Supplementary Figure S4

Expected times to absorption for a 2-taxa system

In the 2-taxa-system, the function $T(x, b)$ is as defined in supplementary methods for a range of advantage terms, taking initial relative abundance b = 0.8. Time to fixation is 100× area under the curve, in each graph. The advantage term α^* ranges from zero to 100.

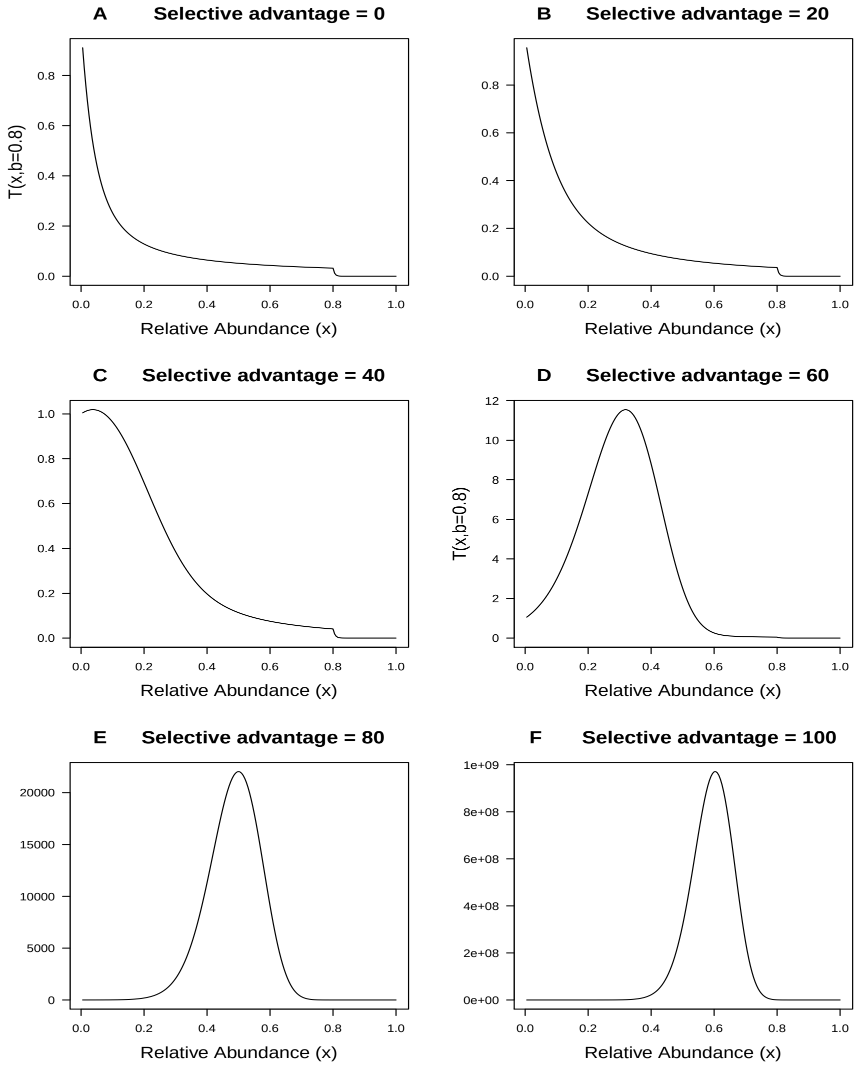

Supplement: S4 Fig — In the 2-taxa-system, the function T(x, b) is as defined in Supplementary S1 Methods for a range of advantage terms, taking initial relative abundance b = 0.8. Time to fixation is 100× area under the curve, in each graph. The advantage term α* ranges from zero to 100. (PDF) [file pone.0117221.s004.pdf]
