# Supplementary material for: Probabilistic Models to Describe the Dynamics of Migrating Microbial Communities
Source: PLoS One. 2015 Mar 24;10(3):e0117221. doi: 10.1371/journal.pone.0117221 (PMC4372544; doi:10.1371/journal.pone.0117221)
Supplement: S2 Table — The average was computed in the same way for both the discrete and continuous simulations. For each of the 50 runs, once the system had settled to a seemingly steady state, we computed the mean relative abundance level and the variability about this mean for each of the taxa. The averages presented here are averages of these quantities estimated from 50 independent simulations when selective advantage (α) is given to the bulk taxon and selective disadvantage of the same magnitude is given to the biofilm taxon. The carrying capacity buffer is not given a selective advantage or disadvantage. (PDF) [file pone.0117221.s006.pdf]

Probabilistic models to describe the dynamics of migrating microbial communities

Joanna L Schroeder, Mary Lunn, Ameet J Pinto, Lutgarde Raskin, William T Sloan

Supplementary Table S6

A comparison of the average mean and variability (after reaching seemingly stable state) for each taxon relative abundance when when selective advantage (α) is given to the bulk taxon and selective disadvantage of the same magnitude is given to the biofilm taxon.

The average was computed in the same way for both the discrete and continuous simulations. For each of the 50 runs, once the system had settled to a seemingly steady state, we computed the mean relative abundance level and the variability about this mean for each of the taxa. The averages presented here are averages of these quantities estimated from 50 independent simulations when selective advantage (α) is given to the bulk taxon and selective disadvantage of the same magnitude is given to the biofilm taxon. The carrying capacity buffer is not given a selective advantage or disadvantage.

α	Carrying Capacity		Bulk Taxon		Biofilm Taxon	
	Discrete	*Continuous*	*Discrete*	*Continuous*	*Discrete*	*Continuous*
0	0	0	0.000 ± 0.002	0	1.000 ± 0.002	1
0.02	0	0	0.013 ± 0.037	0.023 ± 0.047	0.987± 0.037	0.977 ± 0.047
0.04	0	0	0.465 ± 0.081	0.484 ± 0.082	0.535 ± 0.081	0.516 ± 0.082
0.06	0	0	0.644 ± 0.057	0.663 ± 0.054	0.356± 0.057	0.337 ± 0.054
0.08	0	0	0.732 ± 0.043	0.748 ± 0.039	0.268 ± 0.043	0.252 ± 0.039
1	0	0	0.785 ± 0.034	0.799 ± 0.032	0.215 ± 0.034	0.201 ± 0.032
