# Supplementary material for: Probabilistic Models to Describe the Dynamics of Migrating Microbial Communities
Source: PLoS One. 2015 Mar 24;10(3):e0117221. doi: 10.1371/journal.pone.0117221 (PMC4372544; doi:10.1371/journal.pone.0117221)
Supplement: S1 Methods — (PDF) [file pone.0117221.s008.pdf]

Probabilistic models to describe the dynamics of migrating microbial communities

Joanna L Schroeder, Mary Lunn, Ameet J Pinto, Lutgarde Raskin, William T Sloan

Supplementary Methods S8

Theoretical Expected Times to Absorption

To investigate the long run behaviour of the system we consider the simple case when there are two taxa in the system, one which cannot migrate in and the other which can. This system can be described using the one dimensional stochastic differential equation (1). Here we suppose that X is the relative abundance of the taxon which cannot migrate into the system but which is already present (i.e. the 'bulk' taxon). The other taxon (i.e. the biofilm taxon) can migrate in but is at a disadvantage when it does. W is normally distributed random variable with mean zero and unit variance and α^* denotes the advantage term. The initial abundance of the bulk taxon is denoted b throughout.

$$dX = [-mX + \alpha^* X(1-X)]dt + \sqrt{2X(1-X)dt}W \tag{1}$$

From relative abundance b at $t = 0$, the expected time to fixation of the migrating taxon is given by (2) [**?**, **?**]

$$\hat{T}(b) = \int_0^1 T(x,b)dx \tag{2}$$

Where the function $T(x, b)$ is

$$\begin{aligned} T(x,b) &= \frac{1}{x}(1-x)^{m-1}\exp^{\alpha x}\int_0^x (1-y)^{-m}\exp^{-\alpha y}\,dy && \text{for } 0 \le x \le b \\ T(x,b) &= \frac{1}{x}(1-x)^{m-1}\exp^{\alpha x}\int_0^b (1-y)^{-m}\exp^{-\alpha y}\,dy && \text{for } b \le x \le 1 \end{aligned} \tag{3}$$

Expected times to absorption are abtained for values α^* used in simulation, taking the initial relative abundance of the non-migrating taxon to be $b = 0.8$. If 100 units of time corresponds to N^2 individual events ($N = 10^3$), then for $\alpha^* = 0, 20, 40, 60100$ the expected times to fixation are 10, 15, 30, 33.9, 4.5×10^5, 1.6×10^{11} respectively. These parameters are borne out by the simulations involving three taxa, since here we assume the migrating taxon already occupies 20% of capacity and only two taxa are present.
